# FEN1 inhibitor increases sensitivity of radiotherapy in cervical cancer cells

**DOI:** 10.1002/cam4.2615

**Published:** 2019-10-31

**Authors:** Jin‐Li Li, Jian‐Ping Wang, Hong Chang, Sheng‐Ming Deng, Jia‐Hui Du, Xiao‐Xiao Wang, He‐Juan Hu, Dong‐Yin Li, Xiang‐Bin Xu, Wei‐Qiang Guo, Yao‐Hua Song, Zhigang Guo, Min‐Xuan Sun, Yi‐Wei Wu, Song‐Bai Liu

**Affiliations:** ^1^ Department of Radiation Oncology The Affiliated Hospital of Soochow University Suzhou China; ^2^ Suzhou Key Laboratory for Medical Biotechnology Suzhou Vocational Health College Suzhou China; ^3^ Department of Nuclear Medicine The Affiliated Hospital of Soochow University Suzhou China; ^4^ College of Food Science and Technology Hainan University Haikou China; ^5^ School of Chemistry, Biology and Materials Engineering Suzhou University of Science and Technology Suzhou China; ^6^ Cyrus Tang Hematology Center Collaborative Innovation Center of Hematology Soochow University Suzhou China; ^7^ Jiangsu Key Laboratory for Molecular and Medical Biotechnology College of Life Science Nanjing Normal University Nanjing China; ^8^ Jiangsu Key Laboratory of Medical Optics Suzhou Institute of Biomedical Engineering and Technology Chinese Academy of Sciences Suzhou China

**Keywords:** cervical cancer, *FEN1*, radiotherapy, targeted therapy

## Abstract

**Background:**

Cervical cancer is one of the most common causes of cancer‐associated mortality among affected women in the world. At present, treatment with weekly cisplatin plus ionizing radiation (IR) therapy is the standard regimen for cervical cancer, especially for locally advanced cervical cancer. The purpose of this study is to determine whether FEN1 inhibitors could enhance the therapeutic effect of IR therapy.

**Methods:**

Western blot was applied to determine the expression of FEN1‐ and apoptosis‐related proteins. Cell growth inhibition assay and colony formation assay were used to determine the effects of FEN1 inhibitor and IR exposure for Hela cells in vitro. CRISPR technology was used to knockdown FEN1 expression level of 293T cells, and tumor xenograft in nude mice was employed to determine the effects of FEN1 inhibitor and IR exposure on tumor growth in vivo.

**Results:**

Our data revealed that FEN1 is overexpressed in HeLa cell and can be upregulated further by IR. We also demonstrated that FEN1 inhibitor enhances IR sensitivity of cervical cancer in vitro and in vivo.

**Conclusion:**

FEN1 inhibitor SC13 could sensitize radiotherapy of cervical cancer cell.

## Graphical abstract

FEN1 is overexpressed in HeLa cell and the expression can be upregulated further by IR. FEN1 inhibitor enhances IR sensitivity of cervical cancer, and this enhancement effect was largely due to the impairment of DNA damage repair mechanism resulting from FEN1 inhibition, leading to apoptosis of cancer cells.

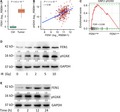

## 1 INTRODUCTION

Cervical cancer is 1 of the top 10 commonly diagnosed and lethal cancers in female worldwide.^1^, ^2^ Radiotherapy has been used as a primary treatment for cervical cancer for many years, especially for locally advanced cervical cancer.^3^, ^4^ Ionizing radiation (IR) can affect DNA structure stability and repair processes by directly interacting with any of the individual DNA moieties, or by indirect interaction with the induced reactive species from molecules surrounding DNA. These DNA lesions include single‐strand breaks (SSB), double‐strand breaks (DSB), and DNA cross‐links.^3^, ^5^, ^6^ If IR‐induced DNA damages are not sufficiently repaired by DNA repair system, cancer cells proceed to genomic instability, apoptosis, and death.^7^, ^8^, ^9^ Cisplatin can binds to two adjacent G residues of DNA and form intra‐strand crosslink, formation of cisplatin‐DNA adducts, resulting in DNA replication and transcription arrest.^10^, ^11^, ^12^, ^13^ Currently, Cisplatin is used as a first‐line therapy for cervical cancer following radiotherapy.^3^, ^14^, ^15^, ^16^ However, patients who initially respond to cisplatin therapy often develop resistance to the drug during subsequent treatment. The potential nephrotoxicity, ototoxicity, and highly emetic effects of cisplatin also limit its use to certain populations.^13^, ^17^, ^18^ Therefore, it is essential to develop radiation sensitizer with high efficiency and low toxicity for the treatment of cervical cancer. Since DNA repair system plays important roles in radioresistance of cancer cells, targeting the DNA damage repair pathways and related genes may offer potential therapeutic advantages to overcome the radioresistance.

DNA flap endonuclease‐1 (FEN1) is a member of RAD2 superfamily nucleases. It plays an essential role in Okazaki fragment maturation of DNA replication, and is an important component in DNA repair pathways such as base excision repair (BER) and polymerase α error editing (AEE) pathway.^19^, ^20^, ^21^, ^22^ FEN1 is reported to be overexpressed in many forms of cancer, and FEN1 inhibitor has been reported to enhance the effect of DNA damage‐related chemotherapy drugs such as cisplatin, 5‐FU, and paclitaxel.^18^, ^23^, ^24^, ^25^

In this study, we determined if FEN1 inhibitor SC13 could sensitize cervical cancer cell to radiotherapy. We demonstrated that FEN1 is overexpressed in HeLa cell and can be upregulated further by IR induction. We also showed that FEN1 inhibitor enhances IR sensitivity of cervical cancer both in vitro and in vivo, and the beneficial effect was largely due to the impairment of DNA damage repair mechanism resulting from FEN1 inhibition, leading to apoptosis of cancer cells.

## 2 MATERIALS AND METHODS

### 2.1 Cell lines and SC13 inhibitor

HeLa cell line was from the American type culture collection (ATCC). The cells were cultured in 90% of DMEM (GE Healthcare Life Sciences) with 10% of fetal bovine serum (Invitrogen), at 37°C in humidified 5% CO_2_ incubator. SC13 inhibitor was synthesized by our laboratory and dissolved in DMSO before use.^24^

### 2.2 Antibodies

Antibodies used in this paper are listed as following: anti‐FEN1 antibody (42 282, Genetex), anti‐γH2AX antibody (ab26350, Abcam), anti‐GAPDH antibody (264 140, Abmart), anti‐BAX antibody (AB026, Beyotime), anti‐BCL‐XL antibody (AB126, Beyotime), anti‐BCL‐2 antibody (AB112, Beyotime), Dy Light 594 Goat‐anti Rabbit (A23420, Abbkine), Dy Light 488 Goat‐anti mouse (A23210, Abbkine).

### 2.3 Western blot

The cells were lysed to extract the total protein using Minute™ Protein Extraction Kits (Invent Biotechnologies) with PMSF. The concentrations of the extracted proteins were quantified using a Bradford Protein Assay Kit (Thermos Fisher Scientific). The samples were denatured by boiling in a water bath at 100°C for 5 minutes. Following incubation with the primary antibodies (anti‐FEN1 antibody (1:500), anti‐γH2AX antibody (1:500), anti‐GAPDH antibody (1:1000), anti‐BAX antibody (1:500), anti‐BCL‐XL antibody (1:500), anti‐BCL‐2 antibody (1:500)), the membranes were incubated with an HRP‐conjugated secondary antibody (Dy Light 594 Goat‐anti Rabbit (1:2000), Dy Light 488 Goat‐anti mouse (1:2000)). The bands were visualized using an enhanced chemiluminescence (ECL) detection system. The protein expression level detected by Western blot was quantified by Image J software. All the experiments were performed three times.

### 2.4 Cell viability assay

Cells were seeded in the 96‐well plates at a density of 3000 cells per well. After treated with SC13 or IR, they were incubated with 10 μL of CCK‐8 reagent (Dojindo) for 1 hour. The optical density (OD) of each well was measured using a microplate reader at 450 nm, and the OD values are reported as the means ± SD.

### 2.5 gRNA transfection and selection

gRNAs against FEN1 gene (gRNA1: 5′AATGACATCAAGAGCTACTT3′; gRNA2: 5′GAGACCACCAGCCACCTGAT3′) were cloned into pLentiCRISPRv2 plasmid and cotransfected into 293T cells with Lipofectamine 2000 reagent per manufacturer's instruction. After 48 hours, the cells were selected by puromycin (1 µg/mL) for 2 weeks. A small fraction of the puromycin‐resistant cells was then harvested and FEN1 expression level was determined by Western blot.

### 2.6 Colony formation assay

The cells were seeded at 3000 per well in 6‐well plates and incubated for approximately 14 days at 37°C. The cells were then washed with PBS and stained with 0.05% crystal violet. Stained plates were then washed and dried prior to counting the colonies.

### 2.7 Apoptosis analysis

After treated with SC13 or IR, the cells were harvested, washed, and resuspended in PBS. The cells were then stained with the Annexin V/PI cell apoptosis detection kit (Dojindo) according to the manufacturer's instructions. Cells were analyzed by a flow cytometer (Accuri C6, BD).

### 2.8 Antitumor effect on tumor xenograft in nude mice

Five‐ to 6‐week‐old female nude mice were used in this study were housed and maintained under standard NIH protocol.^24^ HeLa cells (2 × 10^6^) were harvested and suspended in PBS buffer, then diluted with equal volumes of matrigel and injected subcutaneously into the right flank of each mouse. After the cancers were established, SC13 (200 µg) were administered intraperitoneally daily for five consecutive days. The mice was locally exposed IR (10 Gy) on the third day after drug injection. Cancer volume was measured every 6 days in each group, and the volumes were calculated as length * width^2^/2. Mice were euthanized after 30 days.

### 2.9 Statistics analysis

Data obtained from multiple experiments were reported as the mean ± standard deviation (SD). Student's *t* test and ANOVA with multiple testing were performed to determine the statistical significance as appropriate. A value of *P* < .05 was considered statistically significant.

## 3 RESULTS

### 3.1 FEN1 is overexpressed in cervical cancer and upregulated by IR induction

**Figure 1:**
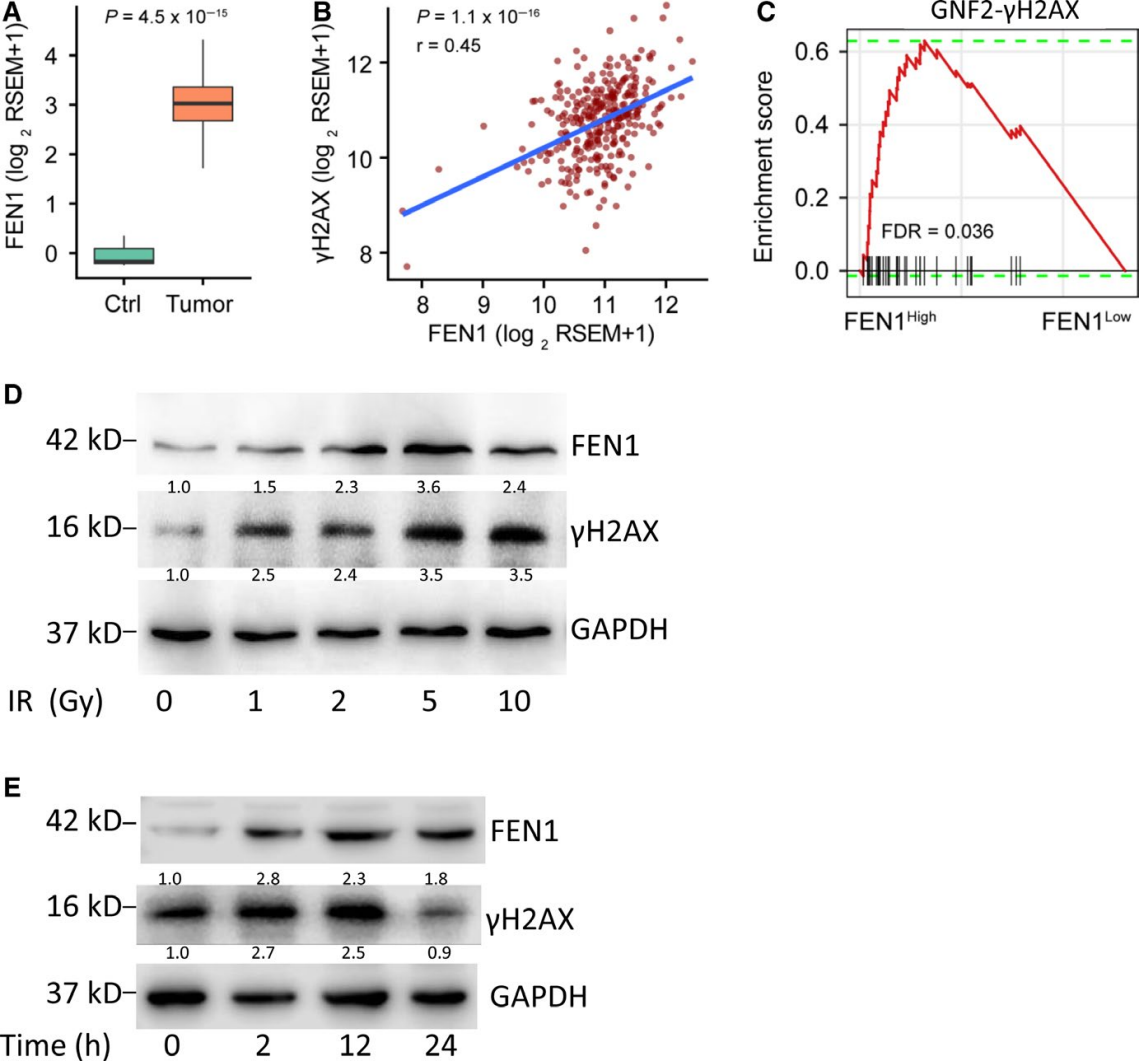
FEN1 is overexpressed in cervical cancer and upregulated by ionizing radiation (IR) induction. A, Expression of FEN1 in cervical cancer samples (Tumor) vs control tissues (Ctrl), using the TCGA cervical cancer dataset. B, Scatter plots showing expression of FEN1 and γH2AX in cervical cancer samples. C, Cervical cancer samples were stratified based on median FEN1 expression. GSEA shows enrichment of the GNF2_H2AFX signature in FEN1^High^ samples vs FEN1^Low^ samples. D, FEN1 and γH2AX expression levels in HeLa cells were determined after 2 h of IR treatment. E FEN1 and γH2AX expression levels at different time point in HeLa cells were determined after IR (5 Gy) treatment

Based on the Cancer Genome Atlas (TCGA) database, FEN1 expression level was about eightfold higher in cervical cancer samples compared to normal tissues (Figure 1A), and was positively correlated with γH2AX expression, a DNA damage sensor (Figure 1B,C). When cervical cancer line HeLa cells were exposed to IR, FEN1 and γH2AX expression levels were both upregulated in an IR dosage‐dependent fashion at 2 hours (Figure 1D). Moreover, FEN1 and γH2AX expression levels were also upregulated in a time‐dependent manner in response to IR (5 Gy) treatment (Figure 1E).

### 3.2 FEN1 inhibitor SC13 enhances IR sensitivity of the HeLa cancer cell

**Figure 2:**
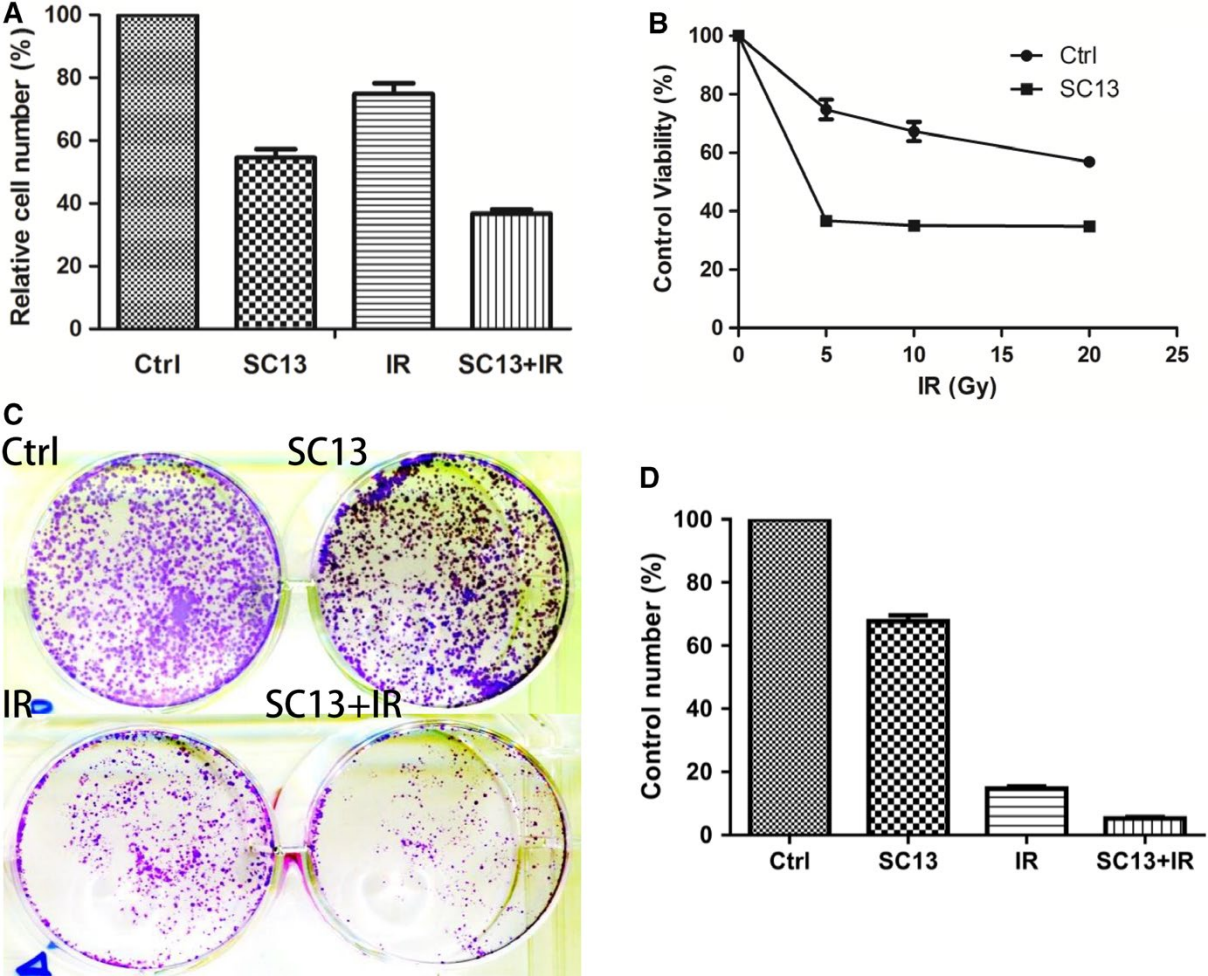
FEN1 inhibitor SC13 enhances ionizing radiation (IR) sensitivity of HeLa cancer cell. A, HeLa cells treated with SC13 (100 µmol/L), IR (5 Gy) alone or combination for 72 h, then determined the cell viability by CCK‐8 kit. B. The survival plots of HeLa cells after IR treatment with or without SC13 (100 µmol/L) incubation. C and D, Colony formation of HeLa cells after treatment with SC13 (40 µmol/L), IR (5 Gy) alone or in combination

Since FEN1 is overexpressed in HeLa cervical cancer cell and upregulated by IR induction, we speculated that the inhibition of FEN1 activity may sensitize IR treatment of HeLa cells. To verify this hypothesis, we incubated HeLa cells with a previously reported FEN1 inhibitor SC13,^24^ in the presence or absence of IR treatment. The results showed that SC13 or IR treatment alone moderately inhibited the viability of HeLa cells, with the survival rate of 54.5% and 74.8%, respectively. However, the combination treatment dramatically inhibited cell viability (*P* < .05) (Figure 2A). Furthermore, with the increase in IR dosage, SC13‐treated HeLa cells became more sensitive to IR than control cells (*P* < .05) (Figure 2B). Colony formation assay also showed that SC13 enhances IR sensitivity of the HeLa cancer cell (*P* < .05) (Figure 2C,D). To confirm these findings, we used CRISPR technology to knockout FEN1 in HeLa cells, however, we failed to obtain FEN1 knockout viable cells after puromycin selection. Then we targeted 293T cell line (normal cell line) and used the same strategy to select FEN1 knockout cells. We performed colony formation assay after IR treatment using the FEN1 knockout cells (Figure S1A,B). The data revealed that cells with FEN1 knockout were more sensitive to the IR treatment than the control cells (*P* < .05) (Figure S1C,D).

### 3.3 SC13 increases IR‐induced cell apoptosis of HeLa cell

**Figure 3:**
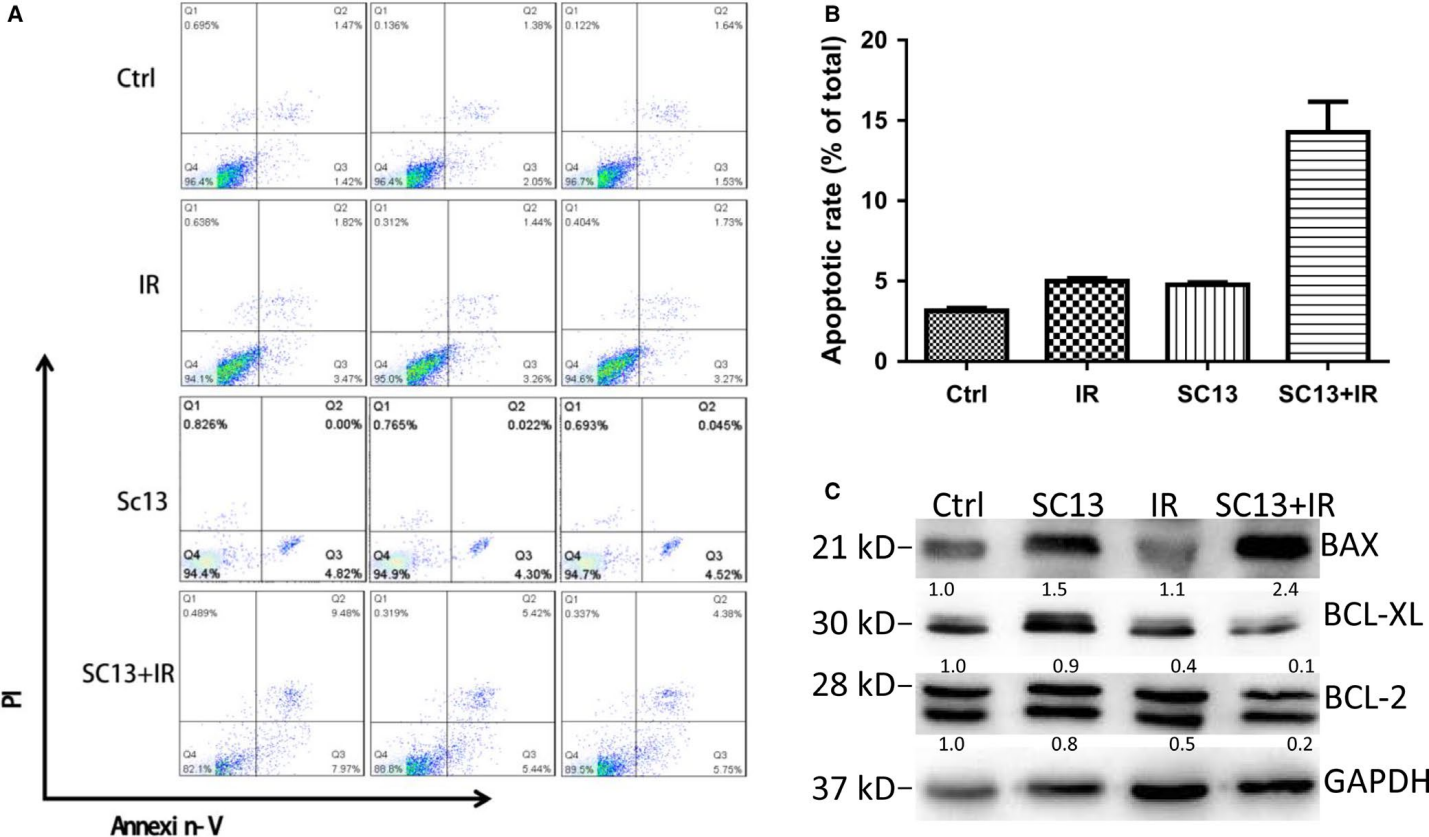
SC13 increases ionizing radiation (IR)‐induced cell apoptosis of HeLa cancer cell. A and B, HeLa cells underwent apoptosis by SC13 (100 µmol/L), IR (10 Gy) alone or combination treatment. C, The expression levels of apoptosis related genes after single or combinative (SC13 [100 µmol/L], IR [10 Gy]) treatment

Radiotherapy can induce cancer cell genomic instability and apoptosis. To determine if SC13 enhances IR‐induced apoptosis of HeLa cells, the flow cytometric analysis was performed using Annexin V/propidium iodide technique. Compared with control cells, the apoptotic rates of IR and SC13 treatment alone were from 3.2% to 5.0% and 4.8%, respectively. However, when IR and SC13 were combined, the apoptotic rate was up to 14.3%, which indicated that SC13 can enhance IR‐induced apoptosis of HeLa cell (*P* < .05) (Figure 3A,B). Anti‐apoptotic BCL‐2 family proteins and proapoptotic family member BAX are apoptosis biomarkers. Western blot analysis revealed that BAX was significantly upregulated in the SC13 + IR treatment group compared with IR or SC13 treatment alone (Figure 3C). By contrast, the expression levels of BCL‐2 and BCL‐XL were lower than that in the IR or SC13 treatment alone group (Figure 3C).

### 3.4 SC13 sensitizes cervical cancer cells to IR in vivo

**Figure 4:**
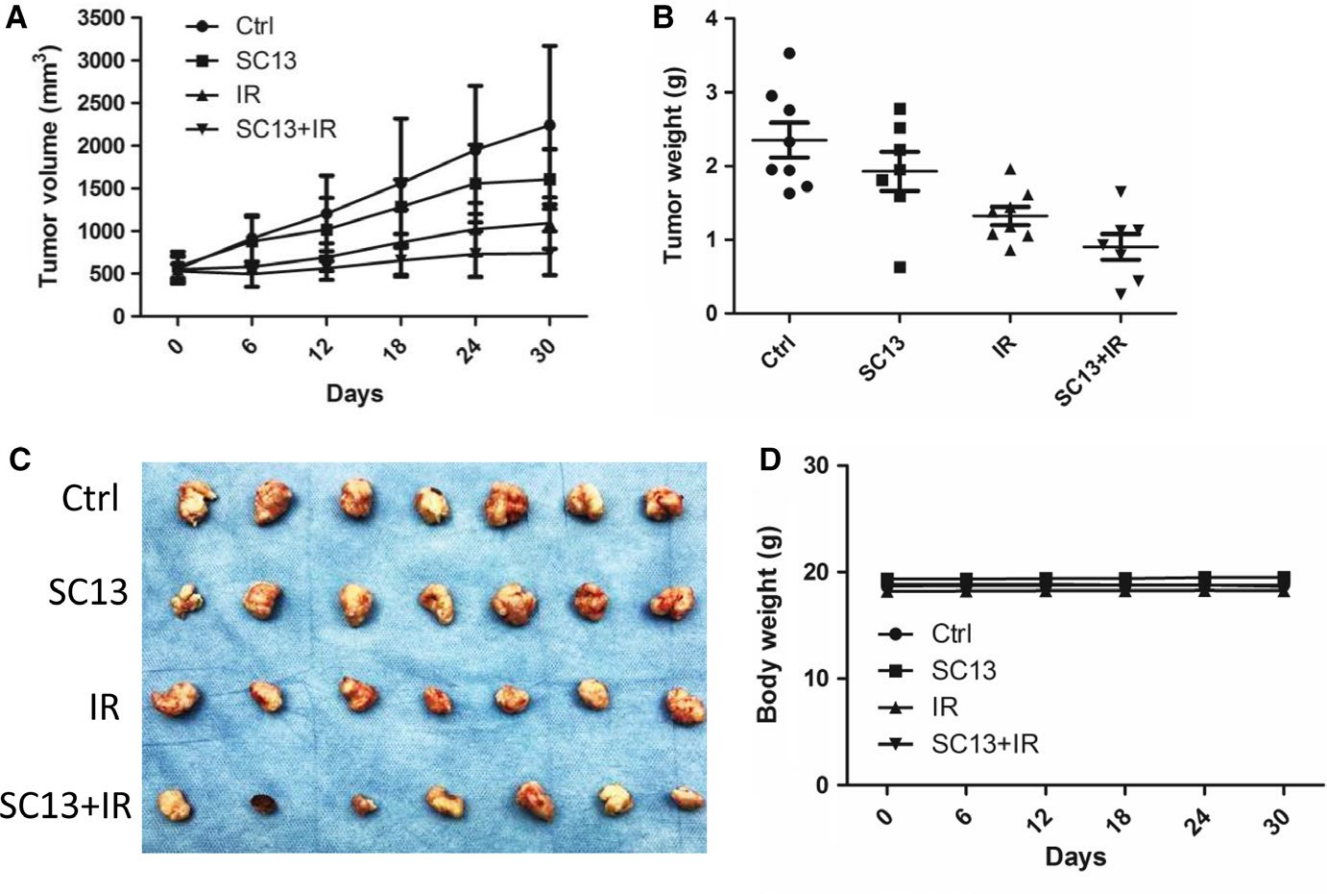
SC13 sensitizes cervical cancer cells to ionizing radiation (IR) in vivo. A, Comparison of tumor progression after treatment with SC13 (200 µg), IR (10 Gy) alone or in combination. B and C, The tumor weight of different treatment group after mice euthanization. D, Body weights of the mice before mice euthanization

To determine if SC13 could sensitize cervical cancer cells to IR in vivo, we performed xenograft experiments using nude mice model. As shown in Figure 4A, tumor cells treated with SC13 or IR alone grown slower than control cancer cells. Cancer cells almost stopped proliferating in the SC13 and IR combination treatment group, and with the slowest rate of growth (*P* < .05). The tumor weights of the mice were also consistent with these results (*P* < .05) (Figure 4B,C). Additionally, the body weights of mice from the four groups were determined and showed no significant difference, which excluded the possible side effect and lethality of SC13 treatment (*P* > .05) (Figure 4D).

## 4 DISCUSSION

In this study, we determined whether FEN1 inhibitor SC13 could enhance the sensitivity of IR treatment in cervical cancer cells. The results showed that FEN1 is overexpressed in HeLa cell and were upregulated further by IR induction. We also demonstrated that FEN1 inhibitor enhanced IR sensitivity of cervical cancer in both in vitro and in vivo models. Since FEN1 plays a vital role in DNA damage repair system, when FEN1 activity is inhibited, damaged DNA induced by IR cannot be repaired efficiently, leading to cell apoptosis. FEN1 is overexpressed in many forms of cancer and has been reported as a potential biomarker and target in different types of cancer. Knockdown of FEN1 could inhibit of proliferation of cancer cells.^18^, ^20^, ^23^, ^26^, ^27^, ^28^ Our data revealed that the growth of HeLa cells was delayed when FEN1 activity was inhibited, which confirmed the previous observation. As an important player in DNA damage repair system, FEN1 is a well‐known enzyme in the BER and AEE pathways, and it is also an important component in other DNA repair pathways, such as nonhomologous end joining and homologous recombination.^29^, ^30^, ^31^ When cancer cells are treated with DNA damage‐related drugs, FEN1 is supposed to be recruited to the damage foci and rescues the damaged DNA in specific sequential order. Indeed, our previous data showed that FEN1 overexpression protected lung cancer cells from apoptosis induced by a DNA damaging drug cisplatin. However, FEN1 deficient lung cancer cells were more sensitive to cisplatin treatment, and leading to more accumulation of unrepaired DNA damages in cells.^18^ Data from breast cancer cell model also verified this conclusion.^24^ IR combines cisplatin treatment is at present a primary treatment for cancer cells, especially for locally advanced cervical and breast cancers; however, patients often develop resistance to the drug during subsequent treatment. In this study, we sought to determine if FEN1 inhibitor could enhance the beneficial effect of IR for cervical cancer. Our results showed that FEN1 inhibitor enhances IR sensitivity of cervical cancer both in vitro and in vivo, which confirmed the conclusion that inhibition of FEN1 can sensitize cancer cells to DNA damage‐related drugs. These studies offer a new strategy to treat advanced cervical cancer in future and lay a foundation for drug development targeting DNA repair proteins. It is of notice that the in vivo synergistic effect of SC13 and IR combination was not as impressive as that of the effect from in vitro data, suggesting that the environments and conditions at cellular level and animal level are somewhat different, and the dosage may need to be further optimized in future studies.

## CONFLICTS OF INTEREST

The authors declare no conflict of interest.

## AUTHOR CONTRIBUTIONS

JL, JW, HC, SD, JD, XW, HH, DL, XX, WG, and YS performed research, analyzed data, and reviewed the manuscript. SL, YW, MS, and ZG designed the research, analyzed and interpreted data, and wrote the manuscript.
