# Supplementary material for: FEN1 inhibitor increases sensitivity of radiotherapy in cervical cancer cells
Source: Cancer Med. 2019 Oct 31;8(18):7774–80. doi: 10.1002/cam4.2615 (PMC6912068; doi:10.1002/cam4.2615)

**Supplemental data**

**Supplemental Figure 1. Knock out *FEN1* expression enhances IR sensitivity of 293T cell.** A. Guide RNAs against *FEN1* gene. B. *FEN1* knock out efficiency detected by western blot assay. C/D. Colony formation of 293T cells after IR exposure of the control group and *FEN1* knock out group.


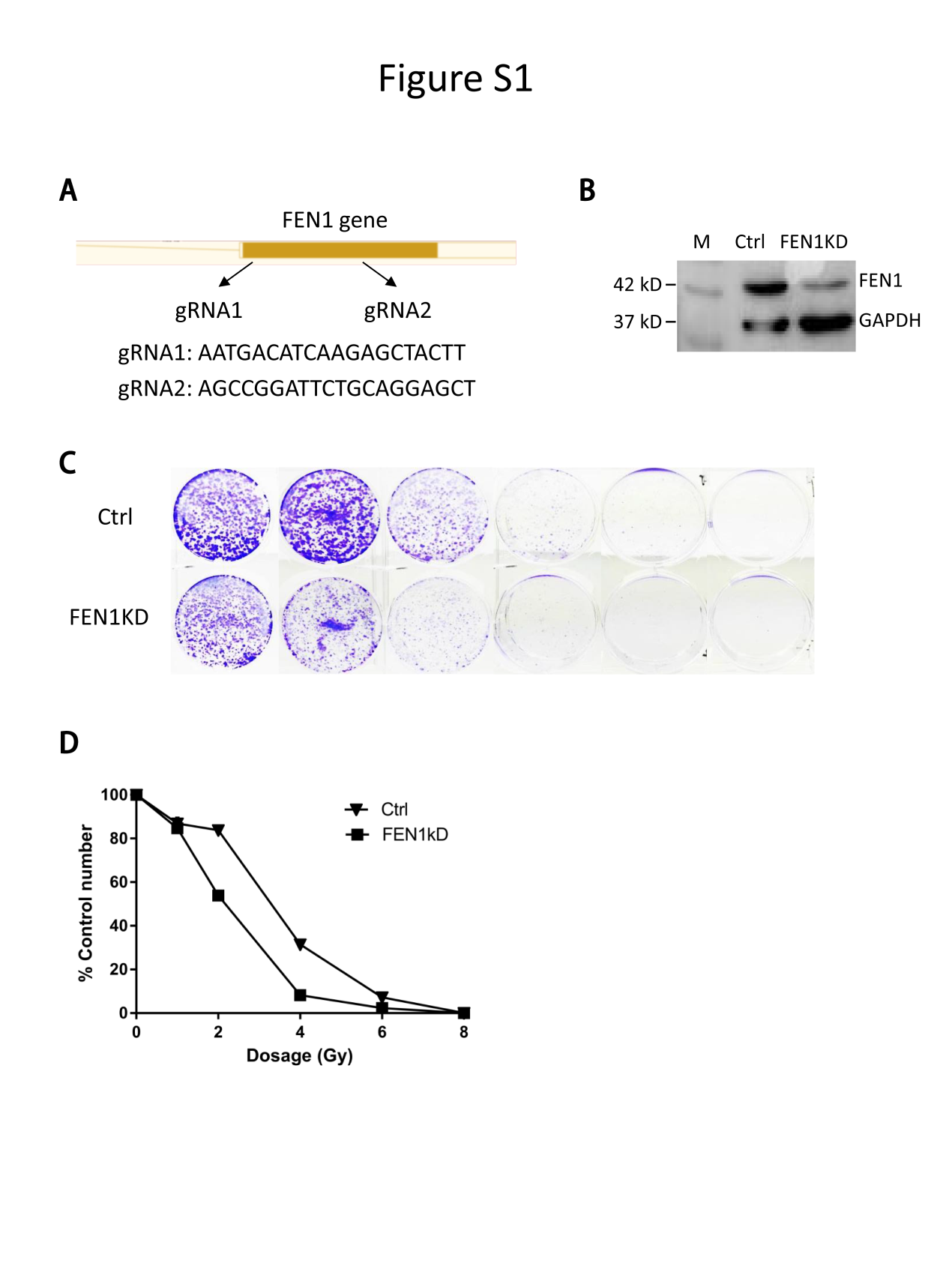

Supplement: Supplementary file 1 [file CAM4-8-7774-s001.docx]
